# Radiobiological evaluation considering the treatment time with stereotactic radiosurgery for brain metastases

**DOI:** 10.1259/bjro.20220013

**Published:** 2022-11-24

**Authors:** Hisashi Nakano, Takeshi Takizawa, Daisuke Kawahara, Satoshi Tanabe, Satoru Utsunomiya, Motoki Kaidu, Katsuya Maruyama, Shigekazu Takeuchi, Kiyoshi Onda, Masahiko Koizumi, Teiji Nishio, Hiroyuki Ishikawa

**Affiliations:** 1 Department of Radiation Oncology, Niigata University Medical and Dental Hospital, 1-757 Asahimachi-dori, Chuo-ku, Niigata-shi, Niigata, Japan; 2 Department of Medical Physics and Engineering, Osaka University Graduate School of Medicine, 1-7 Yamadaoka, Suita-shi, Osaka, Japan; 3 Department of Radiation Oncology, Niigata Neurosurgical Hospital, 3057 Yamada, Nishi-ku, Niigata-shi, Niigata, Japan; 4 Department of Radiology and Radiation Oncology, Niigata University Graduate School of Medical and Dental Sciences, 1-757 Asahimachi-dori, Chuo-ku, Niigata-shi, Niigata, Japan; 5 Department of Radiation Oncology, Institute of Biomedical and Health Sciences, Hiroshima University, 1-2-3 Kasumi, Minami-ku, Hiroshima-shi, Hiroshima, Japan; 6 Department of Radiological Technology, Niigata University Graduate School of Health Sciences, 2-746 Asahimachi-dori, Chuo-ku, Niigata-shi, Niigata, Japan; 7 Department of Neurosurgery, Niigata Neurosurgical Hospital, 3057 Yamada, Nishi-ku, Niigata-shi, Niigata, Japan

## Abstract

**Objective:**

We evaluated the radiobiological effect of the irradiation time with the interruption time of stereotactic radiosurgery (SRS) using CyberKnife^®^ (CK) systemfor brain metastases.

**Methods:**

We used the DICOM data and irradiation log file of the 10 patients with brain metastases from non–small-cell lung cancer (NSCLC) who underwent brain SRS. We defined the treatment time as the sum of the dose–delivery time and the interruption time during irradiations, and we used a microdosimetric kinetic model (MKM) to evaluate the radiobiological effects of the treatment time. The biological parameters, *i.e.* α_0_, β_0_, and the DNA repair constant rate (*a* + c), were acquired from NCI-H460 cell for the MKM. We calculated the radiobiological dose for the gross tumor volume (GTV_bio_) to evaluate the treatment time’s effect compared with no treatment time as a reference. The D95 (%) and the Radiation Therapy Oncology Group conformity index (RCI) and Paddick conformity index (PCI) were calculated as dosimetric indices. We used several DNA repair constant rates (*a* + c) (0.46, 1.0, and 2.0) to assess the radiobiological effect by varying the DNA repair date (*a* + c) values.

**Results:**

The mean values of D95 (%), RCI, and PCI for GTV_bio_ were 98.8%, 0.90, and 0.80, respectively, and decreased with increasing treatment time. The mean values of D95 (%), RCI, and PCI of GTV_bio_ at 2.0 (a+c) value were 94.9%, 0.71, and 0.49, respectively.

**Conclusion:**

The radiobiological effect of the treatment time on tumors was accurately evaluated with brain SRS using CK.

**Advances in knowledge:**

There has been no published investigation of the radiobiological impact of the longer treatment time with multiple interruptions of SRS using a CK on the target dose distribution in a comparison with the use of a linac. Radiobiological dose assessment that takes into account treatment time in the physical dose in this study may allow more accurate dose assessment in SRS for metastatic brain tumors using CK.

## Introduction

Brain metastases are common intracranial malignancies. Approximately, 10–30% of patients with cancer develop brain metastases during the course of their disease, and approximately, 20–30% of these patients die due to poor local control.^1–3^ Non–small-cell lung cancer (NSCLC) is the most common type of brain metastasis, occurring in roughly 40% of cancer patients.^4^ Stereotactic radiosurgery (SRS) has become increasingly important in the management of brain metastases because its use has improved the systemic disease control and reduced the effect on normal tissue.^5,6^ SRS with whole-brain radiotherapy (WBRT) improved local control rates compared to WBRT alone.^7,8^ SRS is an effective treatment for patients with brain metastases from NSCLC.^9,10^

The CyberKnife^®^ (CK) system (Accuray, Sunnyvale, CA) consists of 6 MV flattening filter-free (6 MV-FFF) photon beams produced by a compact linear accelerator (linac) to a multijointed robotic manipulator with six degrees of freedom (6 DoF).^11^ A CK system uses the real-time X-ray exposure to locate the patient’s head between the irradiation, and the linac is controlled by the 6 DoF robotic arm for precise patient positioning based on the results of the tracking algorithm.^12,13^ As a result, changes in the target position are relayed to the robotic arm, which adjusts the pointing of the treatment beam. SRS applied with a CK for brain metastases has shown treatment accuracy and reduces the damage to healthy tissues.^14,15^ It was also reported that compared to conventional (two-dimensional) radiation, the use of a CK in cases such as V12Gy of normal brain in SRS irradiation improved the target dosimetric index (*e.g.* the conformity index [CI]) and reduced the dose to the normal brain compared to a linac.^16^ Therefore, SRS with a CK for brain metastases is an effective radiation protocol in terms of the dose distribution for the target and normal tissue and the patient position accuracy.

The treatment time that is necessary when using a CK system is several times longer than that associated with a linac, since with a CK the prescription dose is irradiated using a large number of non-coplanar beams because the irradiation is performed while correcting the patient positioning during irradiation.^17^ In addition, multiple irradiation interruptions are required for irradiation of the prescribed dose since the CK has a large number of non-coplanar beams and corrects the position of the patient during irradiation to provide precise patient positioning. Thus, the treatment time per fraction is increased in the administration of SRS using a CK.

Several studies have evaluated the radiobiological effects on tumors when the dose–delivery time was prolonged in particle therapy.^18–20^ A large increase in the irradiation time in particle therapy was reported to influence radiobiological effects.^21–23^ The radiobiological effects of photons beams were evaluated when the irradiation time was increased, since a prolonged dose–delivery time and interruption time might reduce the radiobiological effects on tumors.^21–23^ With the use of photon beams, as in the case of particle beams, it is possible to reduce the tumor damage by prolonging the treatment time. However, there has been no published investigation of the radiobiological impact of the longer treatment time with multiple interruptions of SRS using a CK on the target dose distribution in a comparison with the use of a linac. It is important to determine the radiobiological effect of the treatment time for tumors when using a CK, since the treatment time is longer than that of a linac. There have also been no studies that used clinical patient data and irradiation schedules to evaluate the radiobiological effects of the treatment time. We thus propose a dose evaluation method that takes into account the radiobiological effects by considering the treatment time of SRS with a CK for brain metastases.

## Methods

### Patients

We analyzed the cases of 10 patients with 10 brain metastases from NSCLC who underwent brain SRS using a CK during the period from July 2019 to November 2020. Table 2 summarizes the clinical characteristics of the ten patients (all males, median age 70 years, range 63–83 years). The median gross tumor volume (GTV) was 0.72 cm^3^ (range 0.08–2.95 cm^3^). This study was approved by our institutional review board (IRB), and all patients provided their informed consent under our IRB concerning the use of their data for this study.

### Treatment planning

Treatment plans were generated using the Accuray Precision Treatment Planning System (TPS) v. 2.0.0.1. The beam size was controlled with a circular collimator corresponding to the GTV diameter with an M6^™^ series CK (Accuray), and a 6 MV-FFF was used. The selected tracking method was 6D skull tracking, and the beam paths were full head path. Table 2 provides the treatment planning information for each patient. The isocentric and non-isocentric beam arrangement technique was used to irradiate at GTV. The dose calculation algorithm was a ray-tracing algorithm with the 1.0 mm high-resolution condition. 22 Gy was prescribed to 95% of the GTV.^24^

### Calculation of the dose-mean lineal energy y_D_ by Monte Carlo simulations with PHITS

**Figure 1.**
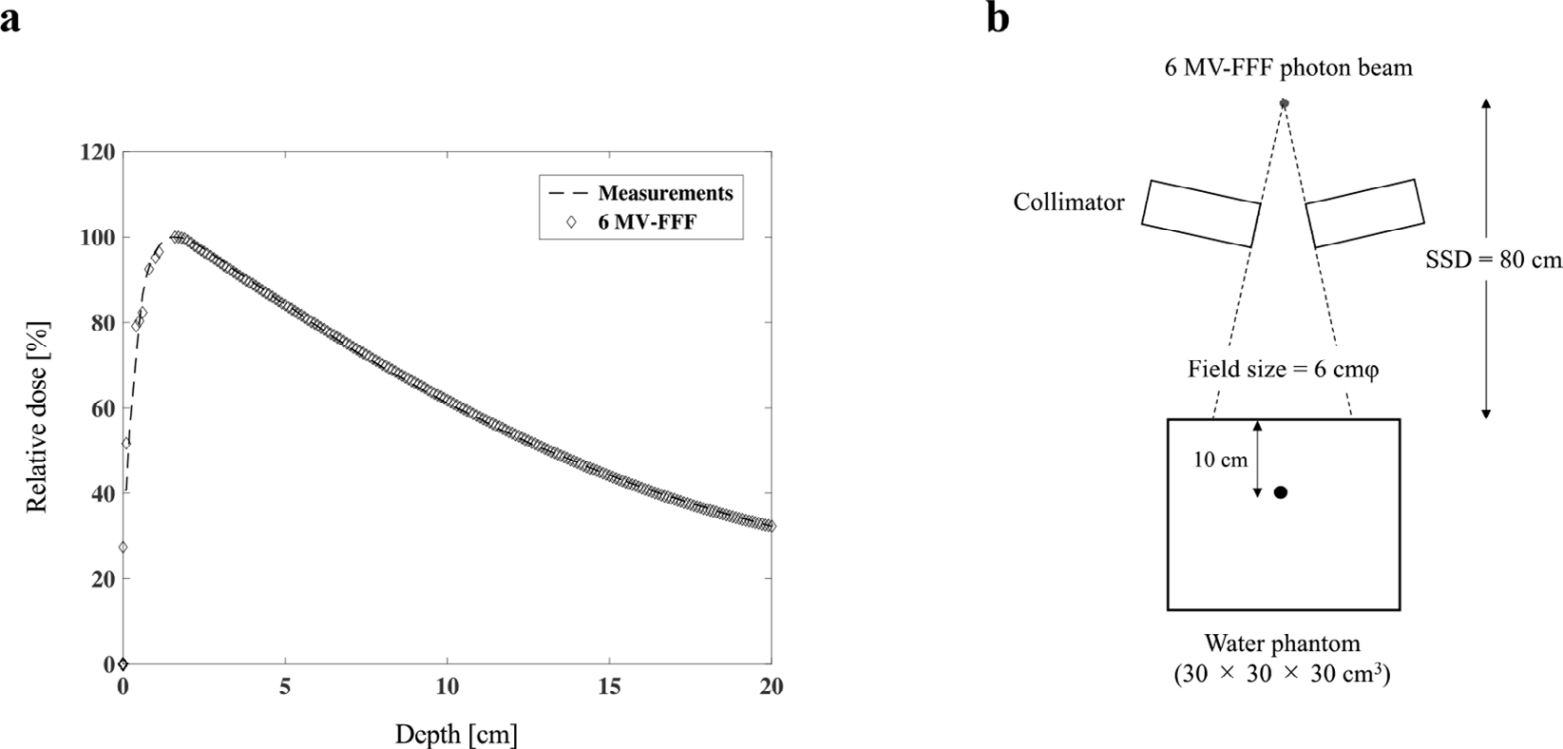
(a) Validation of MC-calculated PDD curves compared with the measurement values by the edge detector for 6 MV-FFF in liquid water. (**b**) Irradiation geometry for the MC calculations with 6 MV-FFF photon beams. The domain radius was 0.5 µm in the 10-cm-deep measurement point in the water-equivalent phantom. FFF, flattening filter-free; MC, Monte carlo; PDD, percentage depth dose.

The percentage depth dose (PDD) of a fixed collimator with a 6 cm beam was modelled using Monte Carlo (MC) simulations. An M6 CK head model fixed collimator was calculated with the BEAMnrc MC simulation system to make the phase space.^25–27^ The number of histories was 1,000,000, and material of collimator was tungsten (W). The photon and electron cut-off energies were set to 0.01 MeV and the MC calculation was performed with a statistical error <1.0%. We transferred the phase-space file created by the BEAMnrc to the particle and heavy ion transport code system (PHITS) v. 3.20 to calculate the dose distribution. The calculated PDD was compared with the measurement value by an edge detector (Sun Nuclear, Melbourne, FL) in a water phantom during the commissioning of the device (Figure 1a).

The lineal energy y was calculated by PHITS v. 3.20. We obtained the dose-mean lineal energy y_D_ value for 6 MV-FFF with 0.5 µm domain radius in the water-equivalent phantom was calculated as a function of y-yd(y): Eqs. (1) to (2).^28–30^:

(1) $$y=\;\frac{\varepsilon }{l}$$

(2) $$y_{D}=\;\frac{\int y^{2}f\left(y\right)dy}{\int yf\left(y\right)dy}=\;\frac{\int yd\left(y\right)dy}{\int d\left(y\right)dy}$$

where ε represents the energy deposited in a domain, *l* is the mean chord length, y is the lineal energy, f(y) is the lineal energy' probability density, and d(y) is the lineal energy’s dose distribution. Figure 1b illustrates the geometry for calculating the y_D_ value. The irradiation geometry was 80 cm source-to-skin distance (SSD), 6 ×  6 cm field size, and a 10-cm-deep measurement point in the water-equivalent phantom (Figure 1b). The calculated y_D_ value was 2.46 ± 0.001 keV/μm for the 10-cm-deep measurement point.

### Relative radiobiological effect with treatment time with a microdosimetric kinetic model (MK model) using irradiation log files

The microdosimetric kinetic (MK) model can be used to calculate the radiobiological effect of the treatment time with photon beams.^31–34^ In the present study, the treatment time (T) was defined as the sum of the photon beam delivery time plus the beam-to-beam interruption time. The MK model can calculate the interruption time for both the beam pulse interval and each field interval. The equation of the MK model is:

(3) $$-l_{n}S=\sum_{n=1}^{N}\left[(\alpha_{0}+\gamma \beta_{0})D_{n}+\beta_{0}D_{n}^{2}\right]\;+2\sum_{n=1}^{N-1}\sum_{m=n+1}^{N}\{\beta_{0}[e^{-(m-n)(a+c)\tau_{n}}]\}D_{n}D_{m}\;=\alpha D+\beta D^{2}$$

(4) $$\gamma =\;\frac{y_{D}}{\rho \pi r_{d}^{2}}$$

(5) $$D=\;\overset{˙}{D}T_{treat}$$

(6) $$\left(a\;+\;c\right)=\;\frac{ln2}{T_{1/2}}$$

We defined the interruption time of each n_th_ field as τ_n_ [minutes (min)], and the D_n_ (D_m_) was defined as the absorbed dose in the n_th_ (m_th_) field at a regular interval [Gy]. Table 1 showed the calculation parameters for the MK model. We used the biological parameters of the human NSCLC cell line NCI-H460 to determine the MK model parameters such as the α_0_, β_0_ values.^35–37^ The parameter ρ is the domain’s density, and r_d_ is the domain' radius (0.5 µm). The variable y_D_ is the dose-mean lineal energy [keV/μm], $\dot{D}$ is the dose rate [Gy/min], and T_treat_ is the treatment time [min]. In addition, the (*a* + c) value was the semicontinuous low-dose-rate teletherapy (SLDR) rate, and deduced by Eq. (6).^38,39^ We used another two cell-specific values (*a* + c) (1.0 and 2.0) to assess the radiobiological effect by varying the DNA repair date (*a* + c) values.^21^

**Table 1.** MKM calculation parameters obtained using NCI-H460 cells

Parameter	Value
α_0_ (*Gy* ^-1^)	0.21 ± 0.16
β_0_ (*Gy* ^-2^)	0.07 ± 0.03
Y_D_	2.46 ± 0.01
*p* (*g/cm* ^3^)	1.00
*r_d_* (μm)	0.50
* $\overset{˙}{D}$ * (μ/min)	1000
*a* + c (*h* ^-1^)	0.46
*T*_1/2_ (h)	1.50

MKM, microdosimetric kinetic model.

The clinical irradiation schedule of photon beams was derived from the irradiation log file output after the end of irradiation by the CK. The log file stored time information such as the number of photon beams, the monitor unit, the time of irradiation, and the time when the photon beams were interrupted (Table 2).

**Table 2.** Characteristics, treatment planning, and treatment time in SRS for 10 patients

Age	
Median (range)	70 (63–83)
GTV (cm^3^)	
Median (range)	0.72 (0.08–2.95)
Prescription dose (Gy)	22
Beam arrangement	
isocentric	3
Non-isocentric	7
Beam number	
Median (range)	47 (31–77)
Monitor unit	
Median (range)	5403 (2971–8160)
Treatment time (min)	
Median (range)	10.68 (6.73–16.32)
Delivery time (min)	
Median (range)	5.15 (2.97–8.16)
Average delivery time per field (sec)	
Median (range)	6.28 (4.49–8.03)
Interruption time (min)	
Median (range)	5.85 (3.21–8.86)

GTV, gross tumor volume; SRS, stereotactic radiosurgery.

**Figure 2.**
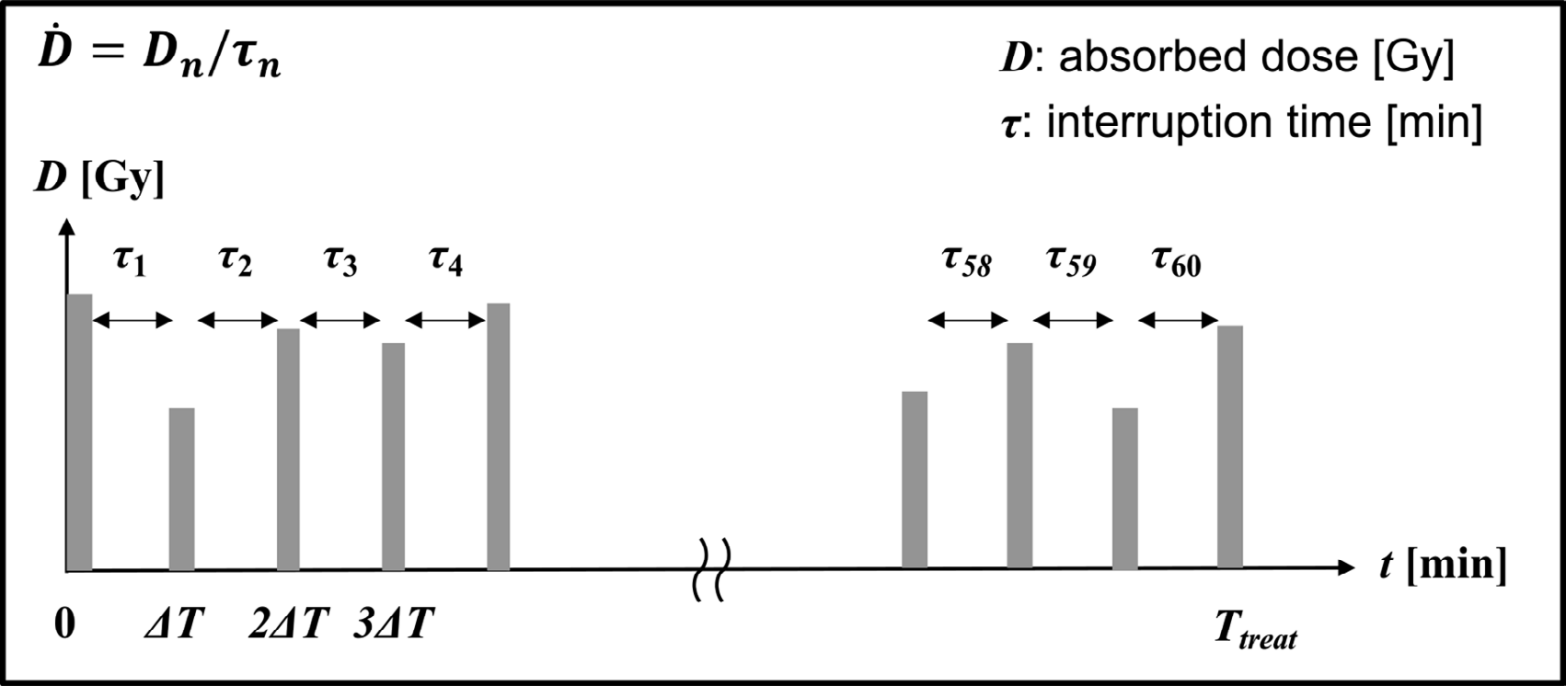
The photon beams' schedules using the MK model considering the photon beams' treatment time using the irradiation log file from CK in Patient 1. CK, CyberKnife^®^; MK, microdosimetric kinetic.

In this study, we defined the treatment time as the time from the first photon beam to the last photon beam based on the time information extracted from the irradiation log files for our evaluation of the radiobiological effects of the treatment time. The surviving fraction was calculated considering the interruption time for both the beam pulse interval and each field interval. Figure 2 illustrates the photon beams' schedules considering the photon beams' interruption time in Patient 1. There were 61 photon beams, and there were 60 interruptions (τ_60_) between the beams (Figure 2). The median treatment time, irradiation time, and interruption time in this study were 10.68 min 5.15 min, and 5.85 min (Table 2).

### Calculation of the time-dependent biological dose effect with the treatment time using the MK model

We defined the time-dependent biological dose effect (TBDE) considering the treatment time by using the physical dose calculated by TPS (treatment time  =  0) with the photon beams as a reference (Eq. 7).^40,41^

(7) $$TBDE=\;\left[\frac{D_{treat=0}}{D_{treat}}\right]=\;{\left(\frac{\sqrt{\alpha_{treat=0}^{2}+\;4\beta_{treat=0}S_{treat=0}}\;-\;\alpha_{treat=0}}{2\beta_{treat=0}}\right)}^{-1}\cdot \;\left(\frac{\sqrt{\alpha^{2}+\;4\beta S}\;-\;\alpha }{2\beta }\right)$$

The TBDE was derived for the dose in the target in each case, and we calculated the relationship between the physical dose of the GTV calculated using the TPS and the TBDE.

## Dosimetric indices with the radiobiological dose (D_bio_) considering the treatment time for GTVs

The radiobiological dose (D_bio_) was calculated taking into account the effects of the treatment time during irradiation by multiplying the physical dose (D_phys_) from the TPS by the TBDE (Eq. 8).^18^ We calculated the D_bio_(x,y,z) by using the TBDE and D_phys_ in all voxels (Eq. 9).

(8) $$D_{bio}=\;TBDE\times D_{phys}$$

(9) $$D_{bio}\left(x,y,z\right)=\sum_{j=1}^{N_{z}}\left[\sum_{i=1}^{Nx,y}\left(TBDE_{ratio\left(i,j\right)}\times D_{phys\left(i,j\right)}\right)\right]$$

The i is the voxel number, N_x,y_ are the number of voxels on the x-y plane, and N_z_ is the number of slices for a CT image. We defined the GTV_bio_ as the volume of the overlapping dose of D_bio_ and the GTV. The dosimetric indices were derived in order to evaluate the effect of the treatment time using the target dose–volume histogram (DVH) of the GTV_bio_ for the GTVs. D95 (%) was evaluated for each GTV with the treatment time. We used the calculated dose distribution to evaluate the conformity of the treatment plans for each GTV. The Radiation Therapy Oncology Group conformity index (RCI) and Paddick conformity index (PCI) were calculated for each GTV with each plan (Eqs. 10, 11).^42,43^

(10) $$RCI=\frac{V_{RX}}{TV}$$

where *V_Rx_* is the volume of the prescription dose and *TV* is the volume of the GTV. The RCI could be evaluated for each target regardless of whether the target volume was over- or undercovered by the prescription volume.

(11) $$PCI=\frac{TV_{PIV}^{2}}{TV\times PIV}$$

where *TV_PIV_* is the target volume within the prescribed isodose, *TV* is the volume of the GTV, and *PIV* is the prescription isodose volume.

## Results

### Treatment time’s effect on the D_bio_ and dosimetric indices for each patient

**Figure 3.**
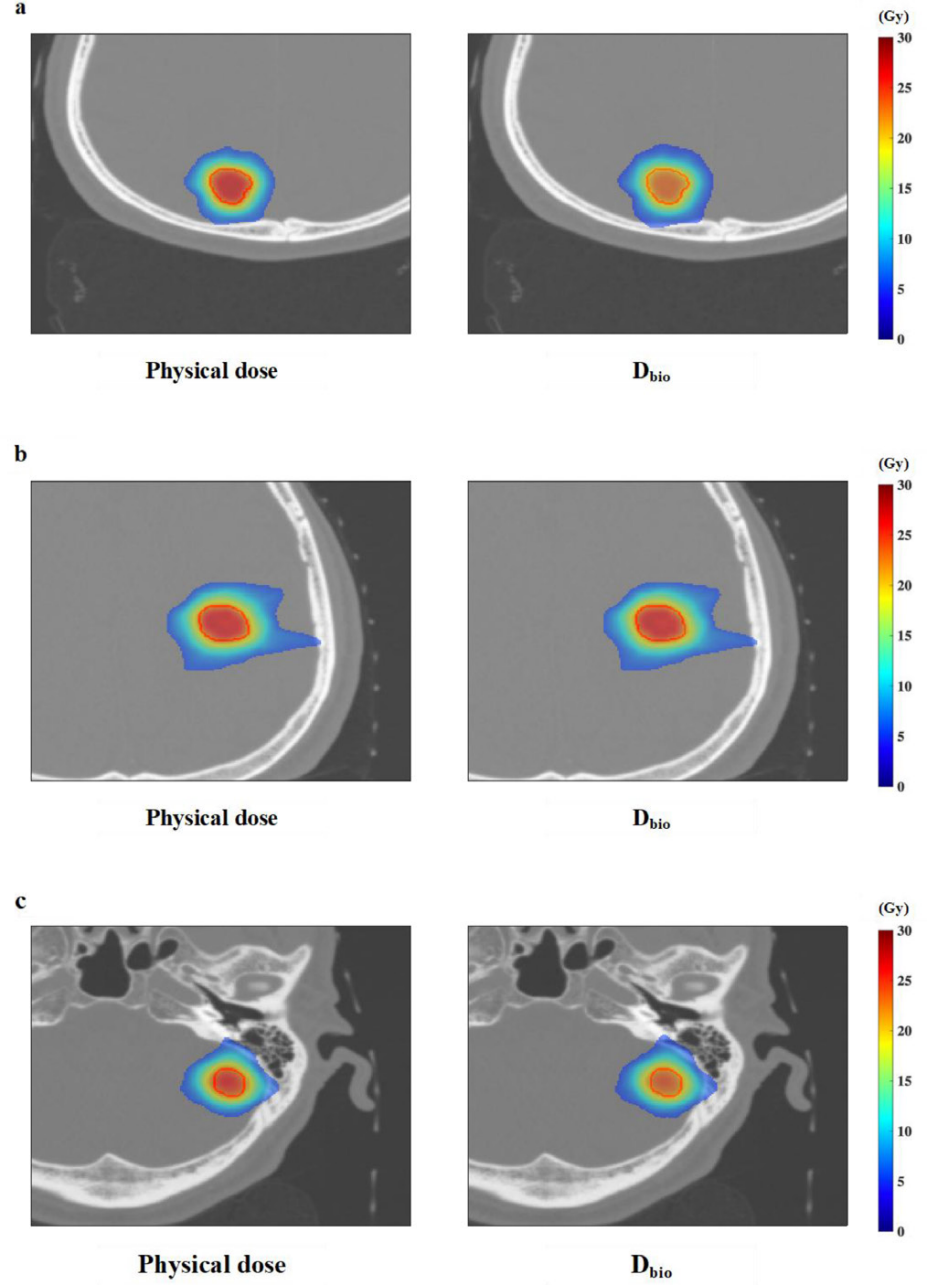
The dose distribution in the axial plane at the isocenter with the physical dose and radiobiological dose (D_bio_) considering treatment time in Patients 1–3 ((**a**)-(**c**)).

We calculated the TBDE for each GTV considering the treatment time from the irradiation log file for the D_bio_. Figure 3 depicts the dose distribution >50% of the prescribed dose for three patients in the axial plane at the center of the GTV with the physical dose from the TPS and D_bio_ considering the treatment time. The physical dose inside the GTV was revealed to be reduced by considering the treatment time.

**Figure 4.**
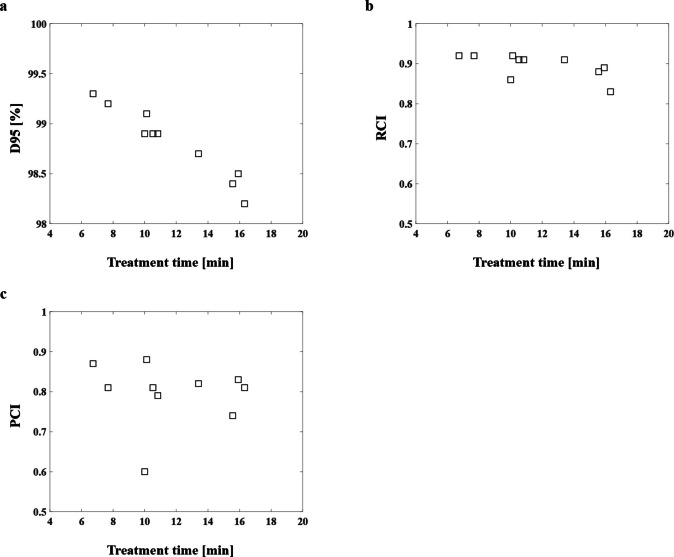
D95 (%), RCI, and PCI considering the treatment time for all patients. PCI, Paddick conformity index; RCI, RTOG conformity index.

We evaluated the dosimetric indices by using the GTV_bio_ (radiobiological dose). The D95 (%), RCI, and PCI considering the treatment time for all patients were shown in Figure 4.

We compared the physical dose for the GTV with 100% D95 (%), 0.95 RCI, and 0.92 PCI, and we observed that the values were decreased to 98.2%, 0.83, and 0.81, respectively when the treatment time was 16.32 min in Patient 1. In Patient 2 (15.92 min) and Patient 3 (10.52 min), the D95 (%) values were 98.4 and 98.9%, respectively (Table 3). The mean values of D95 (%), RCI, and PCI for GTV_bio_ were 98.8%, 0.90, and 0.80, respectively, and decreased with increasing treatment time. The D95 (%) and RCI were decreased with a longer treatment time, with a maximum decrease of 1.8% for the D95 (%) and 0.12 for RCI on the GTV_bio_ (Table 3). The PCI also decreased with prolonged treatment time, showing the largest decrease of 0.16 in patient 9 (Table 3).

**Table 3.** The dosimetric indices considering the effect of treatment time for the GTV for patients

Patient no.	GTV (cm^3^)	Treatment time (min)	D95 (%) (GTV)	D95 (%) (GTV_bio_)	RCI (GTV)	RCI (GTV_bio_)	PCI (GTV)	PCI (GTV_bio_)
1	1.32	16.32	100.0	98.2	0.95	0.83	0.92	0.81
2	2.18	15.92	100.0	98.5	0.95	0.89	0.90	0.83
3	0.73	10.52	100.0	98.9	0.95	0.91	0.84	0.81
4	2.95	10.12	100.0	99.1	0.95	0.92	0.92	0.88
5	0.76	6.73	100.0	99.3	0.95	0.92	0.92	0.87
6	0.41	7.68	100.0	99.2	0.95	0.92	0.83	0.81
7	0.70	13.40	100.0	98.7	0.95	0.91	0.88	0.82
8	0.36	15.57	100.0	98.4	0.95	0.88	0.87	0.74
9	0.08	10.00	100.0	98.9	0.95	0.86	0.76	0.60
10	0.32	10.83	100.0	98.9	0.95	0.91	0.86	0.79
Mean	0.98	11.71	100.0	98.8	0.95	0.90	0.87	0.80
SD	0.92	3.42	0.0	0.40	0.00	0.03	0.05	0.08

GTV, gross tumor volume; PCI, Paddick conformity index; RCI, RTOG conformity index; SD, standard deviation.

### Radiobiological effect of varying the SLDR rate (a+c) values for the GTV_bio_

**Figure 5.**
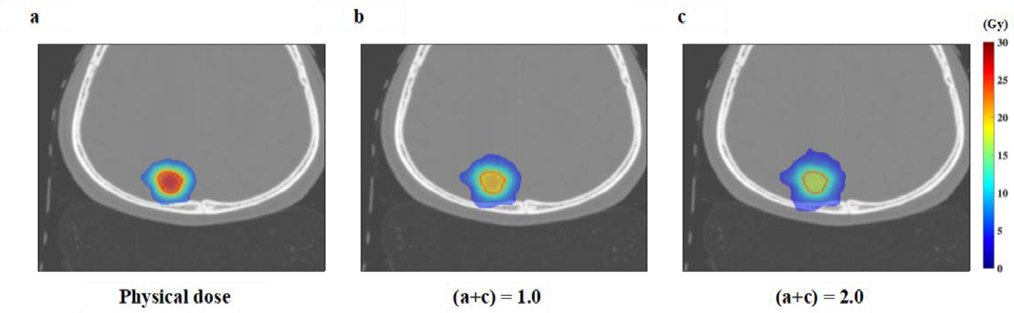
The effect of (*a* + c) on the radiobiological dose D_bio_. (**a**) The physical dose from the TPS as the reference for Patient 1 was (**b**) 1.0 (*a* + c) and (**c**) 2.0 (*a* + c). TPS, treatment planning system.

Figure 5 reveals that the dose distribution >50% of the prescribed dose values in the axial plane at the center of D_bio_ calculated with (*a* + c) were 1.0 and 2.0 in Patient 1.

**Figure 6.**
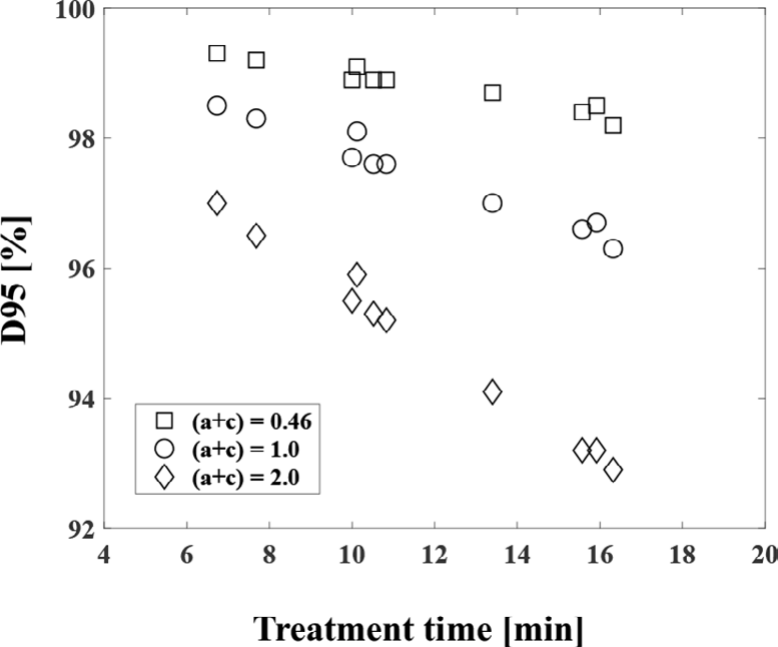
Effect of treatment time on D95 (%) for varying SLDR rate (*a* + c) values for all patients. SLDR, semicontinuous low-dose-rate.

Figure 6 shows the D95 (%) varying the SLDR rate (*a* + c) values for all patients.

Table 4 summarizes the relationship between the SLDR rate and the D95 (%), RCI and PCI for the GTV_bio_. When the (*a* + c) value was 1.0, the D95 (%), RCI, and PCI for the Patient 1 were 96.3%, 0.81, and 0.67, respectively. The mean values of D95 (%), RCI, and PCI for GTV_bio_ were 97.4%, 0.84, and 0.69, respectively, and decreased with increasing treatment time when (*a* + c) value was 1.0.

**Table 4.** The dosimetric indices of GTV_bio_ varying the SLDR rate (*a* + c) values for all patients

Patient no.	D95 (%) (*a* + c) = 1.0	D95 (%) (*a* + c) = 2.0	RCI (*a* + c) = 1.0	RCI (*a* + c) = 2.0	PCI (*a* + c) = 1.0	PCI (*a* + c) = 2.0
1	96.3	92.9	0.81	0.66	0.67	0.44
2	96.7	93.2	0.84	0.69	0.70	0.47
3	97.6	95.3	0.86	0.74	0.69	0.51
4	98.1	95.9	0.90	0.83	0.83	0.69
5	98.5	97.0	0.89	0.83	0.81	0.71
6	98.3	96.5	0.88	0.82	0.74	0.63
7	97.0	94.1	0.83	0.67	0.68	0.44
8	96.6	93.2	0.79	0.57	0.59	0.31
9	97.7	95.5	0.76	0.54	0.47	0.24
10	97.6	95.2	0.85	0.72	0.69	0.49
Mean	97.4	94.9	0.84	0.71	0.69	0.49
SD	0.8	1.5	0.04	0.10	0.10	0.15

GTV, gross tumor volume; SD, standard deviation; SLDR, semicontinuous low-dose-rate.

In addition, the D95 (%), RCI, and PCI for the Patient 1 were 92.9%, 0.66, and 0.44 with 2.0 (*a* + c) value, respectively. The mean values of D95 (%), RCI, and PCI for GTV_bio_ were 94.9%, 0.71, and 0.49, respectively. The (*a* + c) value with a parameter related to the repair time had a significant effect on the D95 (%), RCI and PCI of the GTV_bio_.

## Discussion

We evaluated the radiobiological effect of the treatment time in the application of SRS for brain metastases using a CK. In this study, the maximum treatment time for brain SRS was 16.32 min, and the dosimetric indices D95 (%) and RCI of the GTV_bio_ showed a maximum decrease. The treatment time was shorter than that in the other cases, resulting in a smaller decrease in the dosimetric indices in patient 5 (Treatment time was 6.73 min). In addition, PCI decreases with increasing treatment time in this study, the greatest decrease was seen in Patient 9 with a treatment time of 10 min. The cause is that PCI is as low as 0.76 due to the small size of GTV of 0.08 cm^3^ at the stage of treatment planning. It is considered that the effect of treatment time had a large effect compared to other 9 cases. The longer the treatment time, the greater was the reduction in the GTV_bio_. Other *in vitro* studies have reported very small dose reductions when the treatment time was within 30 min.^44,45^ The treatment time used in the present study was also within 30 min and the results with a small effect on the dose for the GTV were similar; our present findings can thus be considered to be valid. On the other hand, Aiyama et al concluded that the lower the CI was, the lower the local progression rates for brain SRS with 0.65 CI as the threshold.^46^ The PCI considering the treatment time in this study was 0.65 or more in most of the results. However, it was less than 0.65 in Patient 9. In the case of GTVs with small volume, the impact of treatment time on PCI may be significant and may require attention.

**Figure 7.**
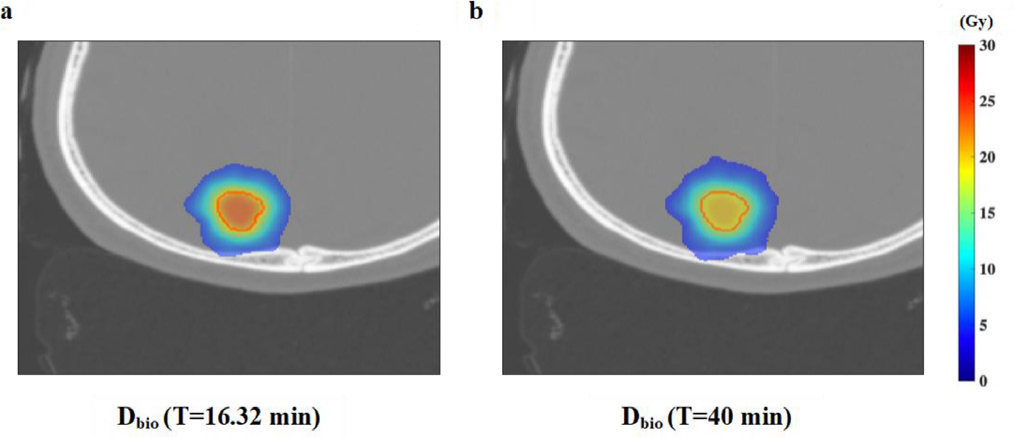
D_bio_ with the treatment times of 16.32 min and the prolonging of the treatment time 40 min in Patient 1.

Treatment times are expected to be longer than those used herein, depending on patient treatment parameters such as prescription dose, target geometry, irradiation technique, and treatment plan to be developed. Han et al investigated treatment times >30 min in SRS for brain tumors using a CK.^16^ Zhang et al reported that irradiation of metastatic brain tumors using the CK requires an average treatment time of 40 min.^46^ Clinical case with treatment times up to 40 min with CK irradiation have been reported, and in such a case, a further decrease in the GTV_bio_ is expected to occur. In the present study, we calculated the GTV_bio_ using Patient 1’s treatment plan information and the average delivery-time per field to assess the effects of a prolonged treatment time. Figure 7 shows the D_bio_ with the treatment times of 16.32 min and 40 min in Patient 1.

The D_bio_ was decreased with the prolonging of the treatment time. The increase in the treatment time affected the dosimetric indices: the D95% decreased from 98.2 to 95.9%, the RCI decreased from 0.83 to 0.79, and the PCI decreased from 0.81 to 0.62. A longer treatment time also resulted in a large GTV_bio_ difference caused mainly by the SLDR. A prescription dose that takes the treatment time into account is thus considered necessary in order to consider more accurate dosimetry to the GTV with a large reduction in the GTV_bio_. If the rate of decrease in GTV_bio_ is large, a way to compensate for the decreased GTV_bio_ may be needed. The GTV_bio_ was decreased in the prescription dose according to the TBDE of each voxel when the treatment time was prolonged. We therefore calculated the compensated dose of the GTV (GTV_comp_) by multiplying (1-TBDE) for each voxel in the GTV by the physical dose in all voxels in the GTV [D_GTV_(x,y,z)]. We calculated the optimal dose (GTV_opt_) that compensates for the dose reduction due to the treatment time by adding the compensation dose GTV_comp_ to the biological dose GTV_bio_ reduced by the treatment time (Eq. 12).

Murphy et al suggested that the treatment delivery staring node in CK treatments when creating the treatment planning should be optimized to minimize the radiobiological effect considering the treatment time.^47^ Therefore, it is necessary to create a treatment plan that completes the treatment as fast as possible when creating the treatment plan. In addition, treatment time should also be as fast as possible during irradiation. There are studies evaluating the impact of total treatment duration extension on tumor overall survival.^48,49^

However, there are no studies evaluating a single treatment time *vs* clinical results, and further data evaluating tumor volume per treatment time is considered necessary. When there is a difference in clinical outcome due to prolonged treatment time, it might be possible to calculate the optimal dose GTV_opt_(x,y,z) considering the treatment time using the relationship between the biological dose D_bio_ and the TBDE of the GTV by optimization in each case when the GTV_bio_ decreases significantly, and irradiated to GTV.

We used NCI-H460 cells to calculate the treatment time’s effect. The D_bio_ was dependent on cell-specific values (*a* + c) of the DNA repair constant rate (Figure 5), and the TBDE was affected by the cell-specific value (*a* + c); the larger that this value was, the greater was the decrease in the GTV_bio_ (Table 4). The GTV_bio_ of the D95 (%) and the PCI were maximally decreased to 92.9% and 0.44 with the treatment time of 16.32 min when the (*a* + c) value was 2.0. In the case of (*a* + c) = 2.0, it was lower than 0.65 CI reported by Aiyama et al under many conditions.^50^ The cell-specific value of the DNA repair indicates the recovery from tumor sublethal damage, depending on the tumor-cell type. Similar to our present results, it has been concluded that the refinement of cell-specific parameters and repair functions are important for building models that take into account biological effects in the current physical models of proton therapy.^18^ Further studies are necessary to evaluate how the treatment time affects specific types of tumor cells in photon therapy.

Several study limitations should be addressed. We simulated the treatment time’s effects by using an MK model and tumor-cell parameters to calculate the biological dose with clinical patient data. It is necessary to compare clinical results assessing how much the reduction in GTV_bio_ due to prolonged treatment time affects tumor volume in order to assess the validity of our present findings. We evaluated the treatment time’s radiobiological effect considering only the tumor SLDR; other repair phenomena such as potentially lethal damage repair and repopulation were not considered. Moreover, the effects of tumor hypoxia and tumor reoxygenation occurring during the treatment time on the GTV_bio_ were not evaluated. Third, the results of this study evaluated only the effect of treatment time on tumor dose in CK and did not compare the results with those of linear accelerators.

## Conclusions

We proposed a dose calculation method that considers the radiobiological effect of the treatment time on tumors when brain SRS for brain metastases is applied using a CK. Cell-specific parameters such as DNA repair constant rate associated with treatment time have a significant impact on radiobiological dose reduction and should be carefully evaluated at the same time.
